# Supporting dataset and methods for Transplacental Transfer of Organochlorine Pesticides: Concentration Ratio and Chiral Properties

**DOI:** 10.1016/j.dib.2019.104278

**Published:** 2019-07-17

**Authors:** Shanshan Yin, Jianyun Zhang, Fangjie Guo, Lu Zhao, Giulia Poma, Adrian Covaci, Weiping Liu

**Affiliations:** a Ministry of Education Key Laboratory of Environmental Remediation and Ecosystem Health, Institution of Environmental Health, College of Environmental and Resource Sciences, Zhejiang University, Hangzhou 310058, China; b Toxicological Centre, University of Antwerp, Universiteitsplein 1, 2610 Wilrijk, Belgium

**Keywords:** Placental transfer, Organochlorine pesticides, Human biomonitoring, Enantiomeric fractions

## Abstract

The dataset and methods provided in this article supports “Transplacental Transfer of Organochlorine Pesticides: Concentration Ratio and Chiral Properties” [1]. The supplementary data were as follows: 1) the information on pretreatment and instrumental methods.2) the data for concentration in the maternal serum, cord serum and placenta samples and data interpretation. 3) the correlation between the influence factors and the log-transformed concentrations of the chemicals samples. 4) the dataset for transplacental transfer ratio of the OCPs and correlations with influencing factors.

**Table:** Specifications Table

Subject	Chemistry
Specific subject area	Analytical Chemistry. Human Biomonitoring.
Type of data	Tables and figures
How data were acquired	Agilent 7890C-7010 gas chromatography-triple quadrupole mass spectrometry system (GC-MS/MS) in the electron ionization mode (EI mode), and Agilent 7890A-5975C GC-MS working under electron capture negative ion chemical ionization (NCI) mode. The data acquisition was done by Agilent Masshunter Workstation.
Data format	Raw and Analyzed
Parameters for data collection	An aliquot of sample (2 mL for serum samples, ∼5 g for lyophilized placenta) was spiked with TCMX as internal standards (50 ng, IS). 0.5 mL of formic acid, 2.5 mL of ethanol and 10 mL of n-hexane/DCM (1:1, v/v) was then added in for the extraction. The mixture was thoroughly mixed, ultrasonic extracted for ten minutes, followed by centrifugation separation at 2000 rpm for ten minutes. The organic phase was then collected; the ultrasonic extraction procedure was repeated three times. The extract was evaporated under vacuum to ∼1 mL, then cleaned up by column chromatography with pre-activated silica gel and Na_2_SO_4_. Targeted compounds were collected using another portion of 70 mL of n-hexane/DCM (1:1, v/v) elution. The elution was evaporated to near dryness in a GC sample bottle, then reconstituted in 50 μL of n-nonane. Total lipids (TL) in the serum was calculated: TL (g/l) = 1.12 × CHOL + 1.33 × TG + 1.48. The lipid weight of the placenta was determined gravimetrically.
Description of data collection	Matched maternal serum, cord serum and placenta samples from 79 volunteer mothers and their infants were collected between November 2015 and March 2016 in Wuhan, China. Informed consent was given by all participants to collect the physical and sociodemographic data. Organochlorine pesticides (OCPs) concentrations were measured in the samples. Enantiomeric fractions were also determined for chiral OCPs in the samples.
Data source location	Yin, Shanshan; Zhang, Jianyun; Guo, Fangjie; Zhao, Lu; Poma, Giulia; Covaci, Adrian; Liu, Weiping (2019), “Data for: Transplacental Transfer of Organochlorine Pesticides: Concentration Ratio and Chiral Properties”, Mendeley Data, V1 https://doi.org/10.17632/dx6ffmm8zn.1
Data accessibility	The data are given with the article and stored in Mendeley Data.
Related research article	S. Yin, J. Zhang, F. Guo, L. Zhao, G. Poma, A. Covaci et al., Transplacental transfer of organochlorine pesticides: Concentration ratio and chiral properties, Environ Int. 130 (2019) https://doi.org/10.1016/j.envint.2019.104939.

**Table:** 

**Value of the Data** • The data in this article presents information on evaluation of pre-treatment, and data interpretation and correlation information for the OCPs in the maternal serum, cord serum and placenta in sampling population. • The data can provide the readers for understanding the transplacental efficiency and related mechanism for the OCPs in human. • The dataset was an expansion in the database of human biomonitoring data in maternal serum, cord serum and placenta in human, also could provide the reference data in the meta-analysis for the cross-membrane investigations.

## 1 Data

**Fig. 1:**
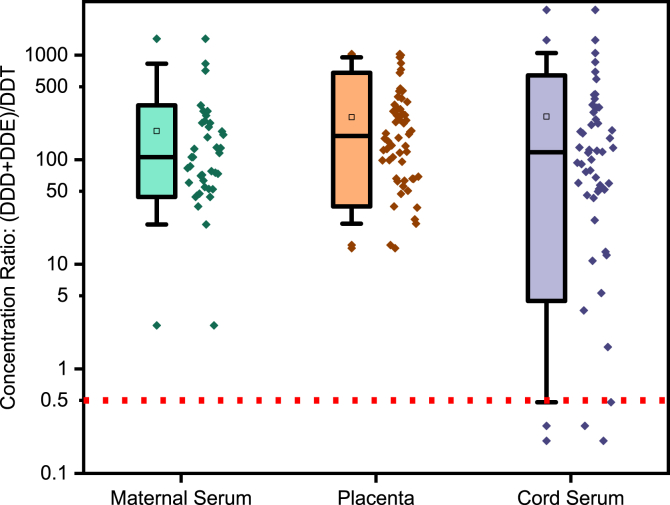
Box-plots with dots representing the lipid adjusted concentration ratio of DDX metabolites (DDD + DDE) *vs*. the DDT parent compounds. The ratio >0.5 reflected historical use of DDT while <0.5 indicated the recent exposure to DDT products. Each dot represents a sample. The box represents the 10th, 50th and 90th percentile of the value, the whiskers represent 5th and 95th. The scale of y-axis was log_10_-transformed.

**Fig. 2:**
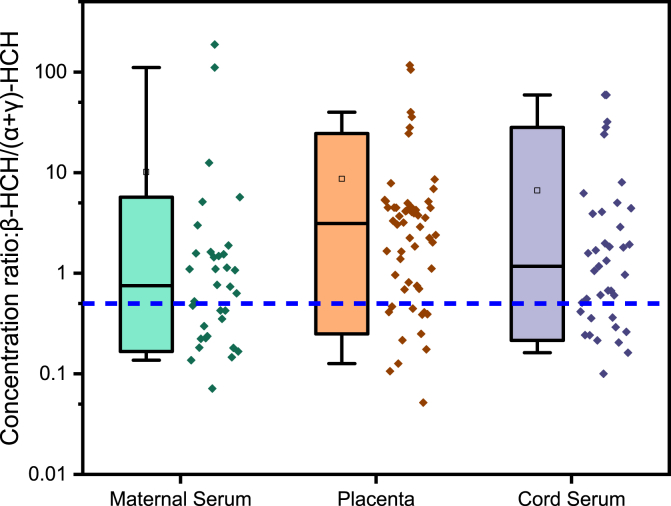
Box-plots with dots representing the lipid adjusted concentration ratio of β-HCH *vs*. (α+γ)-HCH. The ratio ≥0.5 reflected historical use of technical HCHs or lindane while <0.5 indicated the recent exposure to lindane products. Each dot represents a sample. The box represents the 10th, 50th and 90th percentile of the value, the whiskers represent 5th and 95th. The scale of y-axis was log_10_-transformed.

The data reported here provided essential information for the article by Yin et al. [1]. Raw data for Table 1, Table 2, Fig. 1, Fig. 2 were provided in Mendeley Data [2].

**Table 1:** Matrix spiking recoveries (n = 3) at 3 different concentration levels and method detection (MDL) and quantitation limits (MQL) for targeted analytes in serum and placenta samples. The MDL and MQL were defined as the concentration that has 3 or 10 in the signal to noise ratio in the blank matrix.

Serum								
Analytes	Spike recovery ± SD (%)						MDL	MQL
	20 ng mL^−1^		5 ng mL^−1^		0.5 ng mL^−1^		(pg mL^−1^)	(pg mL^−1^)
α-HCH	77.5	±5.2	81.1	±7.7	85.8	±2.9	0.8	2.7
β-HCH	103	±3.4	106	±1.5	96.3	±4.8	3.1	10.4
γ-HCH	93.4	±7.1	102	±6.1	109	±8.0	5.9	19.7
δ-HCH	90.8	±2.8	88.8	±4.5	104	±4.8	4.3	14.4
*o,p’-*DDE	87.3	±6.3	95.6	±7.5	88.3	±2.4	0.6	2.2
*p,p’-*DDE	78.5	±4.6	87.4	±4.8	83.7	±1.8	3.0	9.9
*o,p’-*DDD	92.1	±6.1	107	±6.8	104	±7.4	0.7	2.5
*p,p’-*DDD	86.4	±6.8	92.9	±8.3	92.9	±7.0	0.3	1.1
*o,p’-*DDT	96.8	±3.2	85.5	±7.6	85.1	±2.5	0.4	1.2
*p,p’-*DDT	90.1	±7.1	110.6	±4.7	109	±6.9	0.4	1.5
TCMX^a^	96.3	±3.7	–	–	–	–	–	–
Placenta								

							(pg g^−1^)	(pg g^−1^)
α-HCH	74.3	±5.2	97.6	±2.6	84.3	±6.5	4.1	13.6
β-HCH	86.6	±3.4	112	±5.9	81.6	±1.6	3.9	12.9
γ-HCH	96.6	±7.1	113	±8.8	87.4	±8.0	2.1	7.0
δ-HCH	99.6	±2.8	110	±4.3	82.5	±8.4	1.6	5.3
*o,p’-*DDE	106	±6.3	87.6	±5.4	96.9	±3.7	1.1	3.6
*p,p’-*DDE	109	±4.6	110	±4.7	85.9	±5.8	15.3	51.1
*o,p’-*DDD	92.5	±6.1	109	±7.2	91.6	±7.8	1.2	3.9
*p,p’-*DDD	91.2	±6.8	97.4	±1.0	88.4	±3.0	2.3	7.8
*o,p’-*DDT	75.6	±3.2	80.4	±3.4	86.0	±7.1	1.0	3.4
*p,p’-*DDT	96.3	±7.1	88.5	±2.9	81.1	±1.5	1.1	3.6
TCMX^a^	94.9	±6.8	–	–	–	–	–	–

a TCMX was used as the IS in the experiment, the spike concentration was constant 50 ng in all extraction process in this part of the experiment.

**Table 2:** Detection frequency (DF%) and mean, median (50th percentile) and percentiles of the wet-weight basis concentrations of targeted analytes in maternal serum, cord serum (in ng ml^−1^) and placenta (in ng g^−1^ tissue weight). The concentrations higher than the MDL but lower than the MQL were represented as < MQL in this table.

Analytes	Maternal Serum (n = 52)						Cord Serum (n = 70)						Placenta (n = 57)					
	DF (%)	Mean	SD	25	50	75	DF (%)	Mean	SD	25	50	75	DF (%)	Mean	SD	25	50	75
α-HCH	64	20	11	11	17	26	67	3.4	2.36	<MQL	3.1	4.06	100	102	336	16	29	72
β-HCH	72	336	701	<MQL	84	487	61	72	159	<MQL	14	95	100	1850	3240	157	447	1960
γ-HCH	92	150	417	22.2	40	101	76	27	84	<MQL	8	14	98	663	1610	58.7	187	486
δ-HCH	72	34	15	<MQL	34	41	93	7	4	<MQL	6	9	100	370	968	96.2	188	285
Total-HCHs	100	365	831	40	106	317	100	71	166	9	19	55	100	2980	4230	521	1200	3690
*o,p'-*DDE	63	9	35	<MQL	2	3	73	2	10	<MQL	2.3	8	100	33	61	4	17	35
*p,p'-*DDE	100	434	342	214	335	564	90	145	121	57	113	187	100	4300	6640	864	2300	5870
*o,p'-*DDD	54	3	3	<MQL	2	5	30	<MQL	1	<MQL	<MQL	2.6	91	41	38	11	37	64
*p,p'-*DDD	35	2	2	<MQL	<MQL	3	71	<MQL	1.3	<MQL	<MQL	1.4	72	24	40	<MQL	13	26.5
*o,p'-*DDT	39	3	2	<MQL	2	4	60	<MQL	0.8	<MQL	<MQL	0.8	60	17	29	4	8	18
*p,p'-*DDT	65	3	2	<MQL	2	4	61	4	1	<MQL	1	2	72	35	166	<MQL	7.28	13
Total-DDXs	100	432	347	208	319	590	96	142	125	58	110	192	100	4360	6790	856	2490	6010

Information about the recovery, MDL and MQL for the analytes in the blank matrix of serum and placenta samples were provided in Table 1.

Results of the wet-weight basis concentration of OCPs in samples were provided in Table 2.

Comparison of concentration levels in this dataset and previous researches were listed in Table 3.

**Table 3:** Comparison of OCP concentrations in different regions of the world; (median concentration, presented in ng g^−1^ lipid).

Location of study	n	Total DDXs	*p,p'*-DDT	*p,p'*-DDE	Total HCHs	β-HCH	Refs
**Maternal Serum**							
**Wuhan, China**	**79**	**33.2**	**0.25**	**32.9**	**10.6**	**7.31**	[1]
Shanghai, China	102	408	<LOD	408	138	135	[3]
Bristol, UK	374		11.0	311		47.2	[4]
Yancheng, China	247		7.46	333		27.3	[5]
Taiyuan, China	71	266	17.0	231		73.9	[6]
Korea	147	67.4	5.31	63.4	8.57	7.91	[7]
Spain	308		18.8	243		25.4	[8]
Delhi, India	30		1.4	2.1		5.9	[9]
**Cord Serum**							
**Wuhan, China**	**79**	**32.2**	**0.25**	**31.5**	**5.34**	**4.33**	[1]
Shanghai, China	102	260	<LOD	260	105	97	[3]
Yancheng, China	247		<LOD	193		13.3	[5]
Taiyuan, China	60	169	<LOD	148		35.2	[6]
Korea	117	64.6		63.3	8.47	5.42	[7]
Spain	308		33.3	175		16.9	[8]
Delhi, India	30		0.9	1.7		4.0	[9]
**Placenta**							
**Wuhan, China**	**79**	**15.1**	**0.046**	**15.1**	**7.59**	**3.25**	[1]
Shanghai, China	102	45	<LOD	43	28	28	[3]
Denmark	43	48.9	0.66	47.2	12.0	9.67	[10]
Finland	43	22.27	0.3	21.23	9.64	5.05	[10]
Delhi, India	30		1.9	3.9		8.5	[9]
Korea	108	38.5	3.1	33.1	13.4	12.1	[7]

n: sample number. <LOD: lower than limit of detection (LOD). Bold font markes the supported study by authors.

The Pearson correlation analysis among three different human biological matrices were provided in Table 4.

**Table 4:** Pearson correlation of log-transformed concentration between maternal serum, cord serum and placenta samples.

		α-HCH		β-HCH		γ-HCH		δ-HCH		*o,p'-*DDE		*p,p'-*DDE		*o,p'-*DDD		*p,p'-*DDD		*o,p'-*DDT		*p,p'-*DDT	
		M	C	M	C	M	C	M	C	M	C	M	C	M	C	M	C	M	C	M	C
Lipid-weight based concentration																					
C	n	27		38		42		48		42		50		12		32		12		22	
	β	**.74**^b^		**.98**^b^		**.96**^b^		**.70**^b^		**.79**^b^		**.94**^b^		**.94**^b^		**.97**^b^		**.96**^b^		**.87**^b^	
P	n	32	34	38	38	41	41	47	52	43	52	48	50	26	18	42	38	9	19	33	35
	β	.27	.25	**.96**^b^	**.96**^b^	**.54**^b^	**.46**^b^	**.34**^a^	**.39**^b^	.28	**.33**^a^	**.43**^b^	**.50**^b^	**.53**^b^	**.88**^b^	.15	.02	.40	**.52**^a^	.06	–.10
Fresh-weight based concentration																					
C	β	**.62**^b^		**.97**^b^		**.96**^b^		**.71**^b^		**.79**^b^		**.92**^b^		**.90**^b^		**.96**^b^		**.96**^b^		**.84**^b^	
P	β	**.35**^a^	.26	**.95**^b^	**.94**^b^	**.51**^b^	**.40**^b^	**.28**^a^	**.33**^a^	.23	**.32**^a^	**.42**^b^	**.49**^b^	**.42**^a^	**.83**^b^	.11	–.00	.34	**.50**^a^	–.04	–.12

M: maternal serum, C: cord serum, P: placenta. The correlation was only calculated when the concentration of the chemical was >MDL in both samples. Bold font markes the significant correlations.

a Correlation is significant at the 0.05 level (2-tailed).

b Correlation is significant at the 0.01 level (2-tailed).

The Pearson correlation coefficient (β) between the log-transformed concentrations of the chemicals and the sociodemographic characteristics of the participants in the maternal serum samples, cord serum sample and placenta samples for both lipid-adjusted concentration and wet-weight concentration were provided in Table 5, Table 6, Table 7, respectively.

**Table 5:** Pearson correlation coefficient (β) between the log-transformed concentrations of the chemicals and the sociodemographic characteristics of the participants in the **maternal serum samples**.

	α-HCH		β-HCH		γ-HCH		δ-HCH		*o,p’-*DDE		*p,p’-*DDE		*o,p’-*DDD		*p,p’-*DDD		*o,p’-*DDT		*p,p’-*DDT	
	L	F	L	F	L	F	L	F	L	F	L	F	L	F	L	F	L	F	L	F
Pregnancy weight gain	.21	.26	.00	.02	–.02	–.01	–.41^b^	–.35^a^	–.35^a^	–.34^a^	.00	.04	–.08	–.04	–.11	–.08	.37	.41	.13	.19
Pre-pregnancy BMI	–.24	–.16	.07	.09	.10	.11	.18	.19	.42^b^	.44^b^	.05	.10	.09	.09	–.07	–.05	.09	.10	–.18	–.13
Maternal age	.00	–.01	–.13	–.14	.03	.03	–.17	–.18	.00	.00	–.01	–.02	.21	.23	.01	.01	–.31	–.30	.09	.07
Gestation period	–.06	–.12	–.16	–.19	–.21	–.23	–.40^b^	–.44^b^	.01	–.03	–.04	–.19	.00	–.06	.27	.25	–.23	–.27	–.10	–.16
Occupation	–.13	–.07	.00	.01	.25	.27	.24	.26	–.11	–.09	–.02	–.02	–.04	.02	.24	.26	.07	.08	–.19	–.14
Parity	–.24	–.17	.13	.14	.30^a^	.31^a^	.03	.06	.07	.09	.17	.17	.02	.00	–.02	–.01	–.53^a^	–.55^a^	.27	.28
Abortion	–.07	–.05	.09	.09	.16	.17	.08	.10	.04	.06	.00	–.15	–.08	–.07	–.09	–.09	–.44	–.43	.13	.13
Gestation conditions	.06	.16	.14	.16	.01	.02	.03	.10	.00	.04	.19	.24	.13	.17	.09	.10	.38	.42	.10	.15
Vegetable	.24	.16	–.06	–.08	–.17	–.19	–.11	–.16	.28	.24	–.09	–.18	–.18	–.20	–.22	–.25	.32	.30	–.20	–.26
Meat	–.40^a^	–.29	.12	.15	.12	.16	.30	.35^a^	.14	.19	–.09	.03	.15	.18	–.02	.01	–.26	–.25	.02	.09
Seafood	.00	–.04	–.16	–.18	.18	.16	.07	.02	–.01	–.06	.18	.15	–.34	–.41^a^	.16	.13	.38	.32	–.08	–.15
Drinking water	–.08	–.15	.01	.00	.12	.11	.11	.09	.32^a^	.32	.29^a^	.29^a^	–.25	–.26	.00	–.01	.06	.05	.02	–.01
Tobacco smoking	–.05	.00	.10	.11	.08	.09	–.04	–.02	–.08	–.06	.21	.23	.18	.19	.10	.11	^.^^c^	^.^^c^	.03	.05
Alcohol consuming	–.05	.00	.10	.11	.13	.14	.00	.03	–.02	.00	.06	.09	^.^^c^	^.^^c^	.11	.12	^.^^c^	^.^^c^	.03	.05
Gender of infant	–.23	–.15	–.23	–.22	–.14	–.11	.01	.05	–.01	.00	–.02	–.04	–.07	–.06	–.05	–.03	–.07	–.03	.06	.10

L: Log-transformed lipid-based concentration, F: Log-transformed fresh-weight based concentration.

a Correlation is significant at the 0.05 level (2-tailed).

b Correlation is significant at the 0.01 level (2-tailed).

c Cannot be computed because at least one of the variables is constant.

**Table 6:** Pearson correlation coefficient (β) between the log-transformed concentration of the chemicals and the sociodemographic characteristics of the participants in the **cord serum samples**.

	α-HCH		β-HCH		γ-HCH		δ-HCH		*o,p’-*DDE		*p,p’-*DDE		*o,p’-*DDD		*p,p’-*DDD		*o,p’-*DDT		*p,p’-*DDT	
	L	F	L	F	L	F	L	F	L	F	L	F	L	F	L	F	L	F	L	F
Pregnancy weight gain	–.06	–.11	–.02	–.03	–.03	–.04	–.20	–.22	–.15	–.17	.07	.05	–.03	–.07	–.06	–.07	.14	.12	.12	.10
Pre-pregnancy BMI	.06	.09	.13	.14	.39^b^	.41^b^	.04	.04	.35^b^	.36^b^	.13	.13	.26	.23	.15	.16	.02	.02	.06	.07
Maternal age	.13	.16	–.15	–.15	.08	.10	–.11	–.09	–.05	–.03	.05	.06	.08	.07	–.18	–.18	–.45^b^	–.45^b^	–.15	–.13
Gestation period	–.09	–.13	–.24	–.25	–.20	–.22	–.20	–.22	.01	–.01	–.21	–.22	–.08	–.10	.28	.27	–.11	–.12	–.04	–.05
Occupation	–.07	–.05	–.01	–.01	.14	.15	–.09	–.07	–.08	–.06	.10	.12	.17	.16	.06	.07	.00	.01	–.06	–.02
Parity	–.17	–.16	.22	.21	.22	.23	.19	.17	.04	.03	.04	.04	.22	.16	–.08	–.09	–.29	–.30	.00	.01
Abortion	–.22	–.23	.16	.15	.09	.09	.21	.21	–.07	–.07	–.08	–.08	–.01	–.01	–.15	–.17	–.33^a^	–.34^a^	–.11	–.12
Gestation conditions	.27	.26	.18	.17	.05	.04	.06	.05	.02	.01	.02	.01	.16	.13	.14	.14	.33^a^	.32^a^	.01	–.02
Vegetable	.05	–.02	–.03	–.05	–.10	–.13	.00	–.04	.01	–.02	–.20	–.22	–.34	–.39	–.05	–.07	.01	.00	.08	.06
Meat	–.07	–.05	.07	.07	.18	.18	.07	.08	.19	.21	.04	.05	.31	.30	–.12	–.10	–.06	–.03	–.15	–.11
Seafood	–.20	–.21	–.13	–.14	.12	.12	.07	.06	–.10	–.12	.18	.17	–.13	–.09	.11	.11	.13	.13	.05	.04
Drinking water	–.04	–.07	–.01	–.02	.17	.15	.12	.09	.11	.09	.16	.13	.10	.07	–.10	–.11	–.03	–.04	.03	.01
Tobacco smoking	.09	.05	.14	.13	.08	.09	–.03	–.02	.00	.01	.18	.18	.21	.19	.11	.10	^.^^c^	^.^^c^	–.18	–.14
Alcohol consuming	.09	.05	.14	.13	.08	.07	.02	.00	.03	.02	.06	.04	.21	.19	.11	.10	^.^^c^	^.^^c^	^.^^c^	^.^^c^
Gender of infant	–.07	–.02	–.24	–.23	–.08	–.06	.10	.13	.01	.03	.02	.05	–.04	–.02	–.04	–.02	.08	.10	.22	.27

L: Log-transformed lipid-based concentration, F: Log-transformed fresh-weight based concentration.

a Correlation is significant at the 0.05 level (2-tailed).

b Correlation is significant at the 0.01 level (2-tailed).

c Cannot be computed because at least one of the variables is constant.

**Table 7:** Pearson correlation coefficient (β) between the log-transformed concentration of the chemicals and the sociodemographic characteristics of the participants in the **placenta samples**.

	α-HCH		β-HCH		γ-HCH		δ-HCH		*o,p’-*DDE		*p,p’-*DDE		*o,p’-*DDD		*p,p’-*DDD		*o,p’-*DDT		*p,p’-*DDT	
	L	F	L	F	L	F	L	F	L	F	L	F	L	F	L	F	L	F	L	F
Pregnancy weight gain	.05	.03	.05	.03	.05	.04	–.10	–.11	–.09	–.09	.01	.00	–.07	–.07	.13	.11	.18	.13	–.01	–.02
Pre-pregnancy BMI	.04	–.01	–.14	–.16	–.03	–.05	–.05	–.09	–.11	–.13	–.17	–.18	.22	.16	–.17	–.19	–.01	–.01	–.19	–.20
Maternal age	.14	.08	–.14	–.16	–.07	–.09	–.12	–.15	–.06	–.09	–.16	–.17	.07	.03	–.03	–.07	–.20	–.19	–.13	–.15
Gestation period	–.17	–.19	–.31^a^	–.32^a^	–.25	–.26	–.26	–.28	.01	–.03	–.20	–.20	–.13	–.15	–.12	–.14	–.25	–.20	–.12	–.14
Occupation	–.06	–.05	–.03	–.03	–.05	–.04	.18	.17	–.06	–.05	–.11	–.09	.08	.08	.07	.05	–.18	–.21	–.10	–.08
Parity	–.18	–.22	.10	.05	–.03	–.07	.13	.08	.00	–.04	–.04	–.08	.03	–.03	–.11	–.13	–.08	–.13	.01	–.04
Abortion	–.29^a^	–.28	.05	.04	–.18	–.17	.01	.01	–.05	–.05	–.21	–.19	–.14	–.14	–.15	–.13	–.35	–.32	–.11	–.10
Gestation conditions	–.02	.01	.07	.09	.11	.12	–.02	.01	.13	.14	.04	.06	.24	.24	.07	.09	.06	.09	.23	.23
Vegetable	.08	.04	.07	.03	.08	.04	.15	.11	.01	–.03	.14	.10	.03	–.02	.08	.04	.27	.17	.12	.08
Meat	–.14	–.19	–.05	–.09	–.02	–.06	–.02	–.07	.09	.04	–.05	–.09	.08	.03	–.10	–.14	.01	.00	–.09	–.13
Seafood	–.15	–.10	.00	.02	–.02	.00	–.01	.01	.11	.12	–.04	–.01	–.14	–.09	–.22	–.16	–.08	–.02	.03	.06
Drinking water	.06	.05	–.03	–.03	.25	.22	.21	.19	.12	.10	.15	.13	.19	.17	–.06	–.04	.07	.07	.03	.03
Tobacco smoking	–.11	–.07	.09	.11	.04	.06	.12	.14	.08	.10	.12	.13	.18	.20	–.03	.00	.09	.12	.03	.06
Alcohol consuming	–.08	–.05	.05	.06	.01	.03	.00	.02	.07	.08	.05	.06	.14	.16	–.02	.00	.07	.10	.08	.09
Gender of infant	–.25	–.26^a^	–.25	–.27^a^	–.24	–.25	.24	.19	.03	–.01	–.10	–.12	–.03	–.09	–.02	–.05	–.35^a^	–.39^a^	–.07	–.10

L: Log-transformed lipid-based concentration, F: Log-transformed fresh-weight based concentration.

a Correlation is significant at the 0.05 level (2-tailed).

The Comparison of transplacental transfer ratio of the OCPs with previous published literatures were listed in Table 8.

**Table 8:** Comparison of transplacental transfer ratio with previous published literatures. (Median concentration and IQR, presented in ng g^−1^ lipid).

Cord serum to maternal serum, (R_cm_, lipid adjusted)			Placenta to maternal serum, (R_pm_, lipid adjusted),			Cord serum to maternal serum, (R_cm_, fresh weight based)		Location of the study
n	Median	IQR	n	Median	IQR	Median	IQR	
β-HCH								
38	0.63	(0.48,0.79)	38	0.39	(0.26,0.51)	0.21	(0.16,0.30)	Wuhan, China [1]
28	1.00	(0.9,1.2)				0.30	(0.2,0.5)	San Francisco, USA [11]
73	0.76	(0.50,1.07)	75	0.25	(0.16,0.33)	0.22	(0.15,0.37)	Shanghai, China [3]
247	0.70	(0.46,0.94)	48	0.61	(0.36,0.86)	0.34	(0.23,0.46)	Spain [8]
147	0.83^a^	–	–	–	–	–	–	Korea [12]
*p,p'-*DDE								
50	1.07	(0.95,1.19)	48	0.48	(0.29,0.77)	0.38	(0.33,0.48)	Wuhan, China [1]
62	1.10	(1.0,1.3)	–	–	–	0.40	(0.3,0.5)	San Francisco, USA [11]
86	0.66	(0.43,0.89)	83	0.13	(0.07,0.21)	0.23	(0.13,0.33)	Shanghai, China [3]
88	0.70	(0.06,2.19)	–	–	–	–	–	Thailand [13]
304	0.34	(0.11,0.58)	49	0.17	(0.09,0.23)	0.68	(0.52,0.84)	Spain [8]
147	1.13^a^	–	–	–	–	–	–	Korea [12]

n: sample number, IQR: inter-quartile range, from 25th percentile to 75th percentile.

a R_cm_ calculated from mean or median concentration, lipid-basis.

The Pearson correlation coefficients (β) between the log-transformed cord: maternal serum concentration ratios (R_cm_) of the chemicals and the sociodemographic characteristics of the participants in matched samples were provided in Table 9.

**Table 9:** Pearson correlation coefficients (β) between the log-transformed cord: maternal serum concentration ratios (R_cm_) of the chemicals and the sociodemographic characteristics of the participants in matched samples.

	α-HCH		β-HCH		γ-HCH		δ-HCH		*o,p’-*DDE		*p,p’-*DDE		*o,p’-*DDD		*p,p’-*DDD		*o,p’-*DDT		*p,p’-*DDT	
	L	F	L	F	L	F	L	F	L	F	L	F	L	F	L	F	L	F	L	F
Pregnancy weight gain	–.22	–.32	–.23	–.33^a^	.03	–.06	.03	–.06	.17	.12	.18	–.01	.14	–.25	.01	–.09	–.15	–.37	–.10	–.21
Pre-pregnancy BMI	.56^b^	.44^a^	–.18	–.23	.19	.18	–.14	–.15	.05	.05	–.01	–.10	–.23	–.40	.05	–.01	–.05	–.30	.11	.05
Maternal age	.27	.26	.04	.07	.10	.16	–.05	–.01	–.06	–.02	.02	.08	–.33	–.42	–.09	–.12	.32	.44	–.17	–.08
Gestation period	–.09	–.07	–.19	–.05	–.08	–.02	.05	.05	.20	.23	–.03	.25	.70^a^	.49	–.02	.05	–.06	.23	.22	.30
Occupation	–.08	–.10	.05	.00	–.14	–.17	–.26	–.27	.09	.08	.11	.10	–.17	–.48	.03	–.02	–.55	–.45	.05	.07
Parity	.13	.00	.30	.14	.08	.02	.23	.19	.03	.00	–.31^a^	–.21	.23	.02	.17	.03	.53	.38	–.29	–.37
Abortion	–.15	–.18	.42^a^	.28	.03	.00	.18	.16	.03	.01	–.34^a^	.12	–.59	–.35	.11	–.01	.24	.27	.05	–.03
Gestation conditions	.18	–.02	.16	–.01	–.19	–.23	.00	–.09	.09	.03	–.15	–.26	–.04	–.12	.19	.16	.16	–.09	.03	–.17
Vegetable	–.43^a^	–.39^a^	.09	.06	.38^a^	.38^a^	–.06	–.06	–.07	–.03	–.12	.07	–.07	–.36	–.23	–.16	.14	.18	.13	.25
Meat	.13	.02	–.11	–.21	–.10	–.22	–.03	–.09	.06	.01	–.05	–.28	.20	–.11	–.12	–.22	–.22	–.05	.22	.19
Seafood	.04	.07	–.14	–.05	–.12	–.08	–.06	–.02	–.54^b^	–.52^b^	.09	.09	.46	.53	.16	.25	–.19	.05	.48^a^	.51^a^
Drinking water source	.04	.06	–.12	–.11	.14	.12	–.02	–.02	–.15	–.15	–.25	–.24	–.11	–.45	–.14	–.05	.34	.21	–.06	.01
Tobacco smoking	.12	–.02	.23	.08	.03	.01	.03	.02	.01	.01	–.08	–.10	-c	-c	.02	–.10	-c	-c	-c	-c
Alcohol consuming	.12	–.02	.23	.08	–.13	–.22	.05	–.02	.02	–.03	–.07	–.16	-c	-c	.02	–.10	-c	-c	-c	-c
Gender of infant	.07	.04	–.24	–.18	–.11	–.15	.18	.20	.13	.15	.06	.16	.55	.53	.04	.02	.22	.35	.10	.11

L: Log-transformed lipid-based R_cm_ ratio, F: Log-transformed fresh-weight based R_cm_ ratio.

a Correlation is significant at the .05 level (2-tailed).

b Correlation is significant at the .01 level (2-tailed).

c Cannot be computed because at least one of the variables is constant.

Table 10, Table 11 provides the retention time along with the MS parameters for the quantitative and enantiomeric analysis.

**Table 10:** GC-EI-MS/MS MRM settings for targeted analytes in this dataset.

Analytes	Quantifier			Qualifier			RT (min)
	Parent	Product	CE (eV)	Parent	Product	CE (eV)	
α-HCH	217	181	5	181	145	5	11.9
β-HCH	217	181	5	181	145	5	12.4
γ-HCH	217	181	5	181	145	5	12.6
δ-HCH	217	181	5	181	145	5	13.1
*o,p’-*DDE	246	176.2	30	248	176.2	30	16.3
*p,p’-*DDE	246	176.2	30	248	176.2	30	17.1
*o,p’-*DDD	235	165.2	30	237	165.2	20	17.3
*p,p’-*DDD	235	165.2	30	237	165.2	20	18.1
*o,p’-*DDT	235	165.2	30	237	165.2	20	18.2
*p,p’-*DDT	235	165.2	30	237	165.2	20	19.1

**Table 11:** GC Retention time for chiral chemicals in this dataset.

Analytes	(+)-α-HCH	(−)-α-HCH	(+)-*o,p’*-DDD	(−)-*o,p’*-DDD	(−)-*o,p’*-DDT	(+)-*o,p’*-DDT
Retention Time (minute)	19.3	20.2	56.8	57.2	62.5	63.1

Box-plots with dots representing the lipid adjusted concentration ratio of DDX metabolites (DDD + DDE) vs. the DDT parent compounds, and β-HCH vs. (α+γ)-HCH were provided in Fig. 1, Fig. 2, respectively.

## 2 Experimental design, materials, and methods

### 2.1 Sample collection

Matched maternal serum, cord serum and placenta samples from volunteering mothers (n = 79) and their infant was collected between November 2015 and March 2016 in Wuhan, China. Specific inclusion and exclusion criteria were applied in the baseline cohorts. Eligible mothers included those who are planning to deliver at Wuhan No.1 Hospital, with singleton pregnancy, and without apparent clinical symptoms during the gestation period.

The Maternal blood samples (n = 52) from pregnant women were collected within 3 days prior to delivery, and cord blood samples (n = 70) and placenta (n = 57) were collected at delivery following standard aseptic procedure [14]. Most of the births were by vaginal delivery. Within all samples collected, there were 52 pairs of cord and maternal serum, 48 pairs of placenta and maternal serum, and 57 pairs of placenta and cord serum.

The serum sample was immediately separated by centrifugation at 5000 rpm for 10 min after the collection. The samples were frozen under −20 °C in clean glass containers until analysis.

Informed consent was given by all participants to collect the physical and sociodemographic data, including the maternal age, pregnancy weight gain, pre-pregnancy body mass index (BMI), parity, abortions, gestation conditions (hypertension and diabetes), drinking water source, habit in life (smoking tobacco and alcohol consuming), diet preferences (vegetables, meat or seafood), occupation of the mother and the gender and gestation period for the neonates. The ethical protocol was approved by the research ethics committee of Wuhan No.1 Hospital and Zhejiang University.

### 2.2 Instrumental analysis

#### 2.2.1 Instrumental quantification of OCPs

Target chemicals were quantified with an Agilent 7890C-7010 gas chromatography-triple quadrupole mass spectrometry system (GC-MS/MS) in the electron ionization mode (EI mode). Compounds were separated on an HP-5ms Ultra Inert column (30 m × 0.25 mm × 0.25 μm, Agilent J&W, Agilent Inc. Palo Alto, CA, USA). The injector was set at the splitless injector mode at 230 °C and the volume was 2 μL. The initial oven temperature was 80 °C, increase at 10 °C min-1 to 180 °C, held for 5 min, then increase at 20 °C min^−1^ to 220 °C, and 5 °C min^−1^ to 245 °C. Total running time is 22 minutes. Carrier gas helium was set as constant flow at 1.0 mL min^−1^. Collision gas was nitrogen. The transfer line and the Extractor EI *Source* was set at 280 °C. The detector was set in the multiple reaction monitor (MRM) mode. Ion pairs were selected, and optimized collision energy were explored. The quantifier and qualifier, collision energy (CE) and retention time (RT) are shown in Table 10. The Retention time window was set at 0.5 minutes (−0.2 min to +0.3 min) of the reported retention time. The decimal of the mass in the MRM transitions were set according to Agilent manual, the width of the Quads (MS1 and MS2) were all set at UNIT (0.7 mass wide). The peak areas were acquired to quantify the wet-weight basis concentration in human biomatrices using Agilent Masshunter Workstation (ver. B7.01, Agilent Inc. Palo Alto, CA, USA).

#### 2.2.2 Enantioselective analysis for chiral OCPs

The enantioselective analysis of chiral OCPs (α-HCH, o,p’-DDT and o,p’-DDD) enantiomers was analyzed on an Agilent 7890A-5975C GC-MS working under electron capture negative ion chemical ionization (NCI) mode. The enantiomers were separated on a BGB-172 chiral capillary column (30 m × 0.25 mm × 0.25 μm; BGB Analytik AG, Switzerland). The carrier gas was Helium at the flow rate of 1 mL min^−1^. *Regent* gas for CI operation was methane. Injector and detector temperatures were set at 250 °C and 230 °C, respectively. The injector was set at splitless mode, the injection volume is 2 μL. The initial oven temperature was set at 90 °C, hold for 1 min and then programmed at 20 °C min^−1^ to 160 °C, and 1 °C min^−1^ to 190 °C, which was held for 40 min, and finally increased to 225 °C at 25 °C min^−1^ for 40 min's hold. The detector set in a single ion monitor (SIM) mode. The retention times (RT) are shown in Table 11.

#### 2.2.3 Lipid content in the samples

Total cholesterol (CHOL) and triglycerides (TG) were analyzed by a Beckman Coulter AU5800 Biochem analytic station in Wuhan 1st Hospital after the serum separation. Total lipids (TL) in the serum were used for normalizing the concentration of each pollutant. It was calculated as described previously [15]: TL (g/l) = 1.12 × CHOL + 1.33 × TG + 1.48. The lipid weight of the placenta was determined gravimetrically [16].

### 2.3 QA and QC

All labware used for the extraction was rinsed with hexane twice before the experiment. One procedure blank (hexane) was analyzed for each batch of samples (eight for serum, sixteen for placenta) to check for potential background contamination. The matrix spiked recovery was calculated by extracting the spiked matrix with three levels of the native standards spiking (20 ng mL^−1^, 5 ng mL^−1^, and 0.5 ng mL^−1^). Newborn calf serum and a pooled sample of 10 randomly selected placenta were chosen as the reference matrix for three different types of sample.

The method detection limit (MDL) and method quantification limit (MQL) were defined as the concentration that has 3 or 10 in the signal to noise ratio in the matrix, respectively. The concentrations lower than MDL were considered as not detected. All the concentrations reported in this dataset have already been corrected for the recovery. The calibration curve was prepared in a series of concentrations from 0.05 ng mL^−1^ to 500 ng mL^−1^, forced to pass through zero, the R^2^ > 0.998.

The instrumental sensitivity was monitored by injecting a mixture of the native and surrogate standard at 5 ng mL^−1^ for every 20 samples.

### 2.4 Statistics

In this dataset, each data group were checked with Kolmogorov-Smirnov test for normal distribution. The concentration of target compound (Kolmogorov-Smirnov test, p < 0.05) were then log-transformed to achieve normal distribution for further analysis.

The concentration in different human matrices from the sample volunteer were compared with Wilcoxon signed-rank test. The EF factor between two human matrices were compared with One-way ANOVA test. The difference between EF factors with racemic value was investigated using single sample *t*-test. The transfer ratio of same volunteers (R_pc_, R_pm_ and R_cm_) were compared using paired *t*-test.

The Pearson correlation test was used in this dataset to investigate the association among concentrations from different biomatrices and between the potential impact factors and the concentration/transfer efficiency.

The statistical significance was set at *p* < 0.05. All above-mentioned statistics were done by IBM SPSS version 22 (Chicago, IL, USA).
